# Clinical significance of *STING* expression and methylation in lung adenocarcinoma based on bioinformatics analysis

**DOI:** 10.1038/s41598-022-18278-6

**Published:** 2022-08-17

**Authors:** Ze lin, Yu Liu, Peng Lin, Jinping Li, Jinfeng Gan

**Affiliations:** 1 Department of Biochemistry, Shantou University Medical College, Shantou, People’s Republic of China; 2 Guangxi Key Laboratory of Tumor Immunology and Microenvironmental Regulation, Guilin Medical University, Guilin, People’s Republic of China; 3 School of Biomedical Engineering, School of Ophthalmology and Optometry and Eye Hospital, Wenzhou Medical University, Wenzhou, People’s Republic of China; 4 Department of Histology and Embryology, Faculty of Basic Medical Sciences, Guilin Medical University, Guilin, People’s Republic of China; 5 Department of Pathology, School of Medicine, Jinan University, Guangzhou, People’s Republic of China

**Keywords:** Cancer, Biomarkers

## Abstract

The role of stimulator of interferon genes [STING, also known as transmembrane protein 173 (TMEM173)] in various human cancers has begun to emerge. However, the clinical value of STING in lung adenocarcinoma (LUAD) remains elusive. This study aims to elucidate the clinical significance of *STING* expression and methylation in LUAD. Here, through analyzing data from public resources, we found that both the mRNA and protein expression of STING were reduced in lung cancer. Moreover, lower expression of *STING* was associated with a worse prognosis in LUAD, but not lung squamous cell carcinoma (LUSC). Of note, higher methylation of *STING* was found in LUAD and had the potential to distinguish LUAD tissues from adjacent non-tumor lung tissues and correlated with unfavorable outcomes. Furthermore, the methylation of *STING* could serve as an independent prognostic indicator for both the overall survival (OS) and disease-free survival (DFS) of LUAD patients. Additionally, the constructed nomogram exhibited a favorable predictive accuracy in predicting the probability of 1- and 2-year OS. Our findings suggest that the mRNA expression, and especially the DNA methylation of *STING*, have the potential to be prognostic indicators for LUAD patients.

## Introduction

Cyclic GMP-AMP synthase (cGAS)-STING signaling was initially demonstrated as a DNA sensor axis that mediates innate immune responses against DNA viruses^1^. The cGAS-STING pathway is the first line of defense against DNA viruses^2^. The cGAS-STING pathway is activated by virus-derived DNA, resulting in the production of type I interferons (IFNs) and inflammatory cytokines, as well as subsequent antiviral responses^3^. Recently, a growing body of evidence has implicated cGAS-STING signaling in the suppression of initiation and development of various types of tumors, such as colon cancer^4,5^ and glioma models^6,7^. Tumor-derived DNA induced by radiation therapy can activate the cGAS-STING pathway to result in the production of type I IFNs, maturation of dendritic cells (DCs), and triggering CD8^+^ T cells to eliminate tumor cells^8–10^. Knockdown of STING enhances colony formation and viability of gastric cancer cells^11^. Suppression of the cGAS-STING pathway by nuclear paraspeckle assembly transcript 1 (NEAT1) results in the promotion of lung cancer growth, in syngeneic models, via inhibition of cytotoxic T cell infiltration^12^. Additionally, STING has been implicated in the regulation of lung cancer cell mobility^12^. Knockdown of STING also promotes mobility of gastric cancer cells^11^.

In small cell lung cancer, the STING pathway could be activated by Poly ADP-ribose polymerase (PARP) and Checkpoint kinase 1 (CHK1) inhibitors and promotes anti-tumor immunity^13^. In non-small cell lung cancer (NSCLC), the STING pathway has been shown to be associated with immune checkpoint expression and may predict response to immunotherapy^14^. The clinical significance of STING has been demonstrated in some cancer types^11,15,16^. In gastric cancer, STING expression was found to be decreased in tumor tissues, and its reduced level has been positively associated with various clinical features, including tumor size, tumor invasion, lymph node metastasis, and TNM stage, and its downregulation is closely correlated with poor prognosis^11^. Similarly, in hepatocellular carcinoma, STING expression is inversely associated with tumor size, tumor invasion, and TNM stage, and its downregulation predicts poor OS^15^. In breast and colorectal cancer, low STING expression in endothelial cells is correlated with an increased prevalence of lymphovascular invasion^16^. Breast and colorectal cancer patients with low endothelial STING expression have a significantly poorer OS^16^. There are various mechanisms responsible for the deregulation of STING expression^17^, including DNA methylation^12,18,19^. However, the clinical significance of STING expression and its methylation in lung cancer remains elusive.

Lung cancer, which is classified into small-cell and non-small-cell types, remains the leading cause of cancer-related mortality worldwide^20,21^. NSCLC accounts for ~ 85% of all lung cancer and consists of LUAD and LUSC subtypes^22,23^, with LUAD being the most common type of lung cancer^24^. Despite the advances in diagnosis and therapeutic regimen in recent years, the prognosis of LUAD remains particularly poor^25^. Therefore, there is an urgent need to identify robust novel biomarkers for LUAD patients, to improve patient outcomes.

In this study, we set out to determine the expression and methylation status of *STING* in LUAD and analyze its role in predicting oncologic outcomes of LUAD patients, as well as explore the pathways in which STING may be involved. We found that deregulation of both *STING* expression and methylation had a significant impact on the prognosis of LUAD patients, and *STING* methylation had the potential to distinguish LUAD tissues from adjacent non-tumor lung tissues and an independent prognostic predictor for both OS and DFS of LUAD patients. We also developed a nomogram for predicting the 1 and 2-year survival probability for OS of LUAD patients based on a combination of *STING* methylation with other clinical variables. Together, our study suggests that *STING* expression and its DNA methylation may serve as promising prognostic indicators in LUAD patients. The nomogram survival model may predict LUAD patient outcomes.

## Results

### Both mRNA and protein expression of STING are reduced in lung cancer

**Figure 1:**
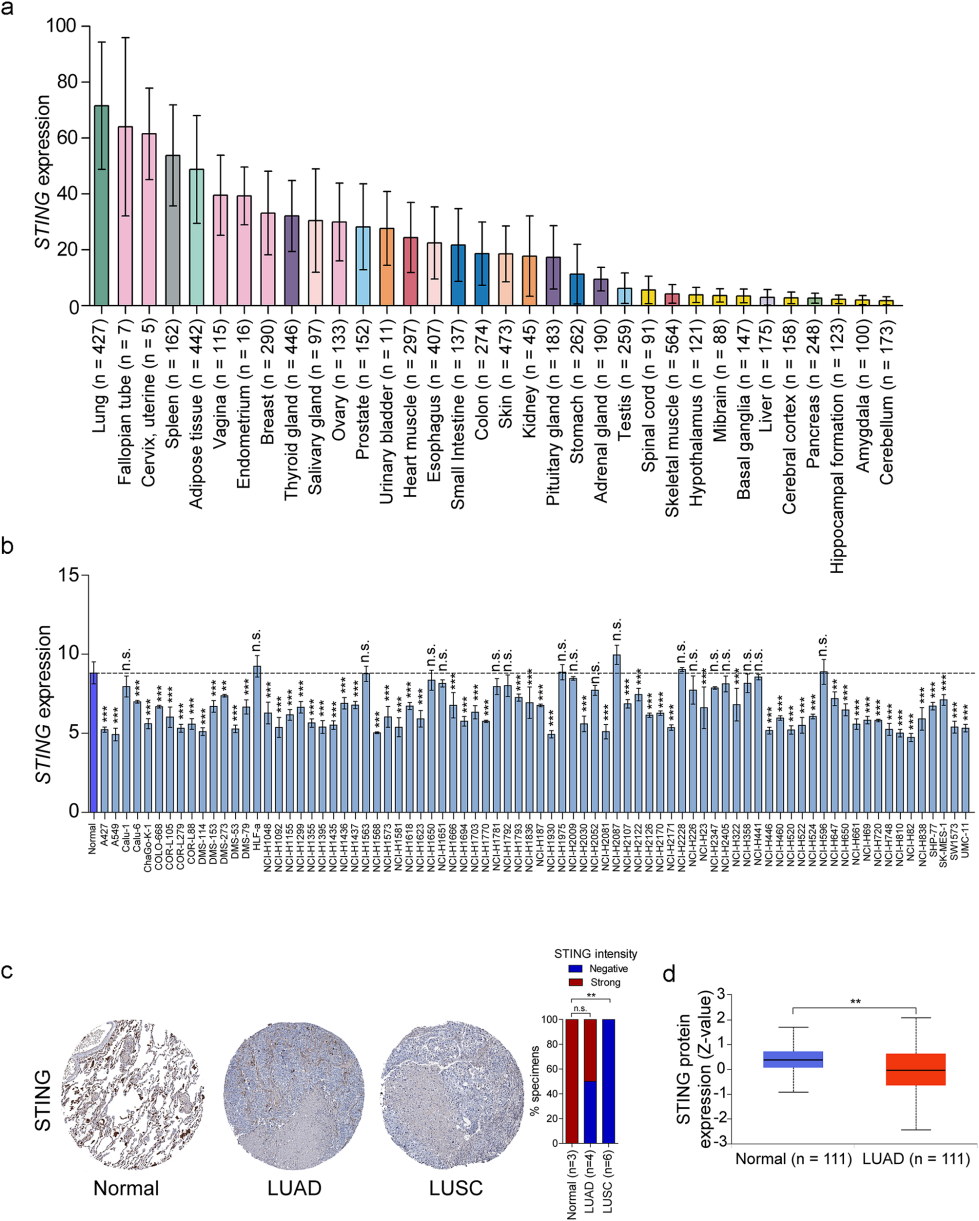
STING is reduced in lung cancer. (**a**) Expression of *STING* in a variety of human tissues was analyzed in GTEx dataset. (**b**) Expression of *STING* in a panel of lung cancer cell lines and normal lung tissues (n = 10) was investigated using the MERAV database. (**c**) Representative immunohistochemistry images of STING staining in LUAD, LUSC, and normal lung tissues were obtained from the Human Protein Atlas database (left panel). The percentage of samples with negative or strong STING staining intensity in LUAD, LUSC, and normal lung tissues was quantified (right panel). (**d**) Protein expression of STING in LUAD was obtained from the UALCAN database. Error bars indicate SD. n.s., not significant. ^**^P < 0.01, ^***^P < 0.001.

To understand the role of STING in cancers, we first obtained the expression data of *STING* across various human tissues from the Genotype-Tissue Expression (GTEx) database^26^, with the access provided by the Human Protein Atlas (https://www.proteinatlas.org/)^27^ We found that lung tissues exhibited the highest level of *STING* expression compared to other normal tissues (Fig. 1a), suggesting *STING* may play a significant physiological role in lung. Therefore, we surveyed STING expression in lung cancer to explore its potential role. Remarkably, 62 of 80 lung cancer cell lines expressed lower *STING* levels when compared to normal lung tissues in the MERAV database (http://merav.wi.mit.edu)^28^ (Fig. 1b). In support of this, in a small cohort of lung cancer patients from the Human Protein Atlas database, STING protein was undetectable in 2 of 4 LUAD patients and 6 of 6 LUSC patients, whereas 3 of 3 normal lung tissues strongly expressed STING protein (*P* > 0.05 for LUAD, *P* < 0.01 for LUSC, Fig. 1c). The difference in STING protein intensity between LUAD and normal lung tissues did not reach statistical significance in Human Protein Atlas database, which may be due to the small sample size. In a larger cohort that consisted of 111 LUAD patients and 111 normal tissues (http://ualcan.path.uab.edu/analysis-prot.html)^29,30^, the significant downregulation of STING protein expression was observed (*P* < 0.01, Fig. 1d). Collectively, these data indicate that STING is decreased in lung cancer at both the mRNA and protein levels.

### Downregulation of STING is correlated with poor outcomes in LUAD patients

**Figure 2:**
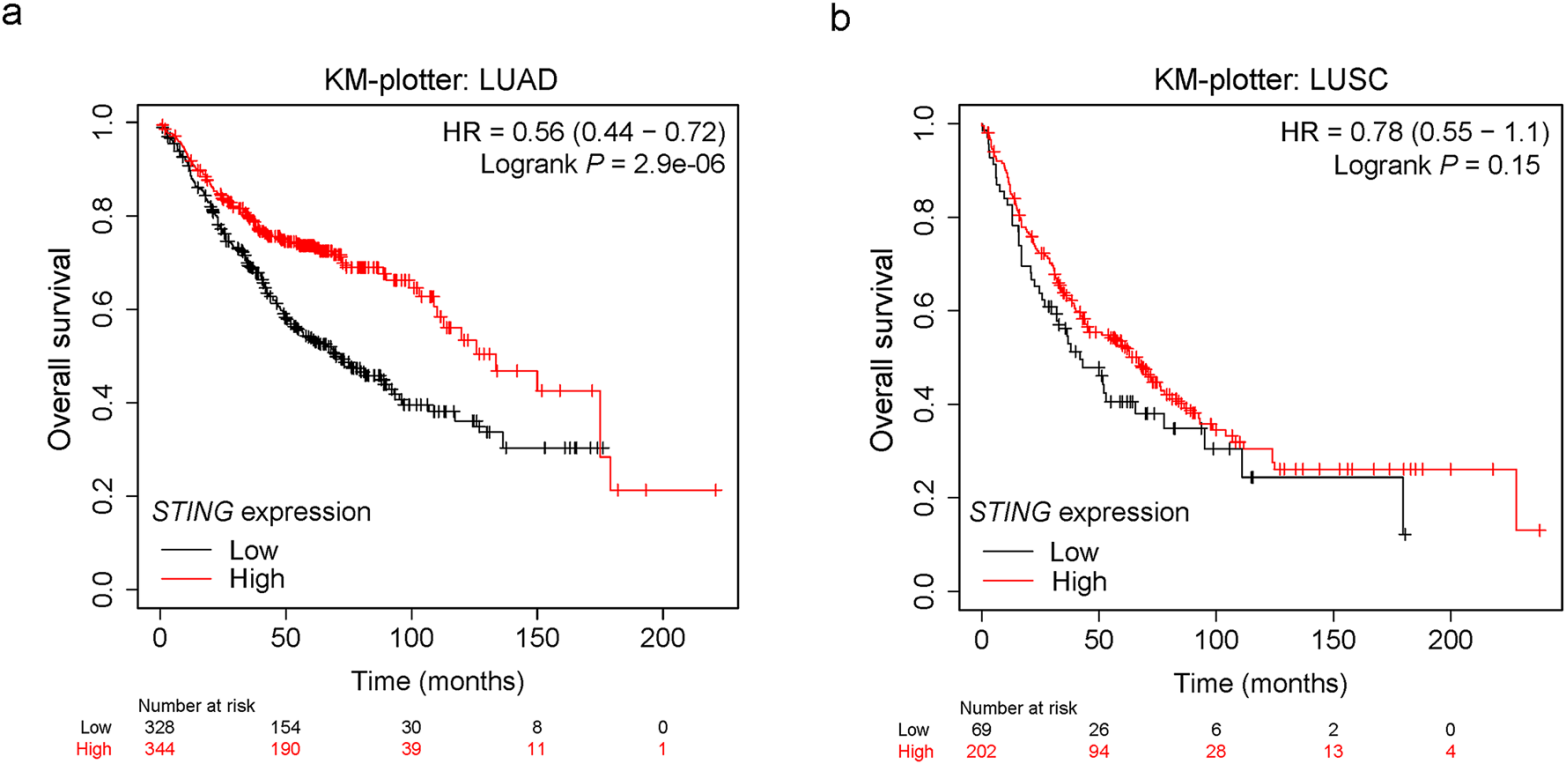
Downregulation of *STING* expression is correlated with unfavorable outcomes in LUAD but not LUSC. (**a, b)** The relationship between *STING* expression and OS in LUAD (**a**) and LUSC (**b**) was investigated using the KM-plotter database. KM-plotter, Kaplan–Meier Plotter.

We next assessed the prognostic role of *STING* in lung cancer, using an online tool (Kaplan–Meier Plotter, http://www.kmplot.com/analysis/index.php?p=service&cancer=lung)^31^. The results showed that patients with low *STING* expression had poorer OS than those with high *STING* expression in LUAD [Hazard ratio (HR) = 0.56, 95% Confidence interval (CI): 0.44–0.72, *P* = 2.9e-06, Fig. 2a). However, we found no significant difference in LUSC patients (HR = 0.78, 95% CI: 0.55–1.1, *P* = 0.15, Fig. 2b).

**Figure 3:**
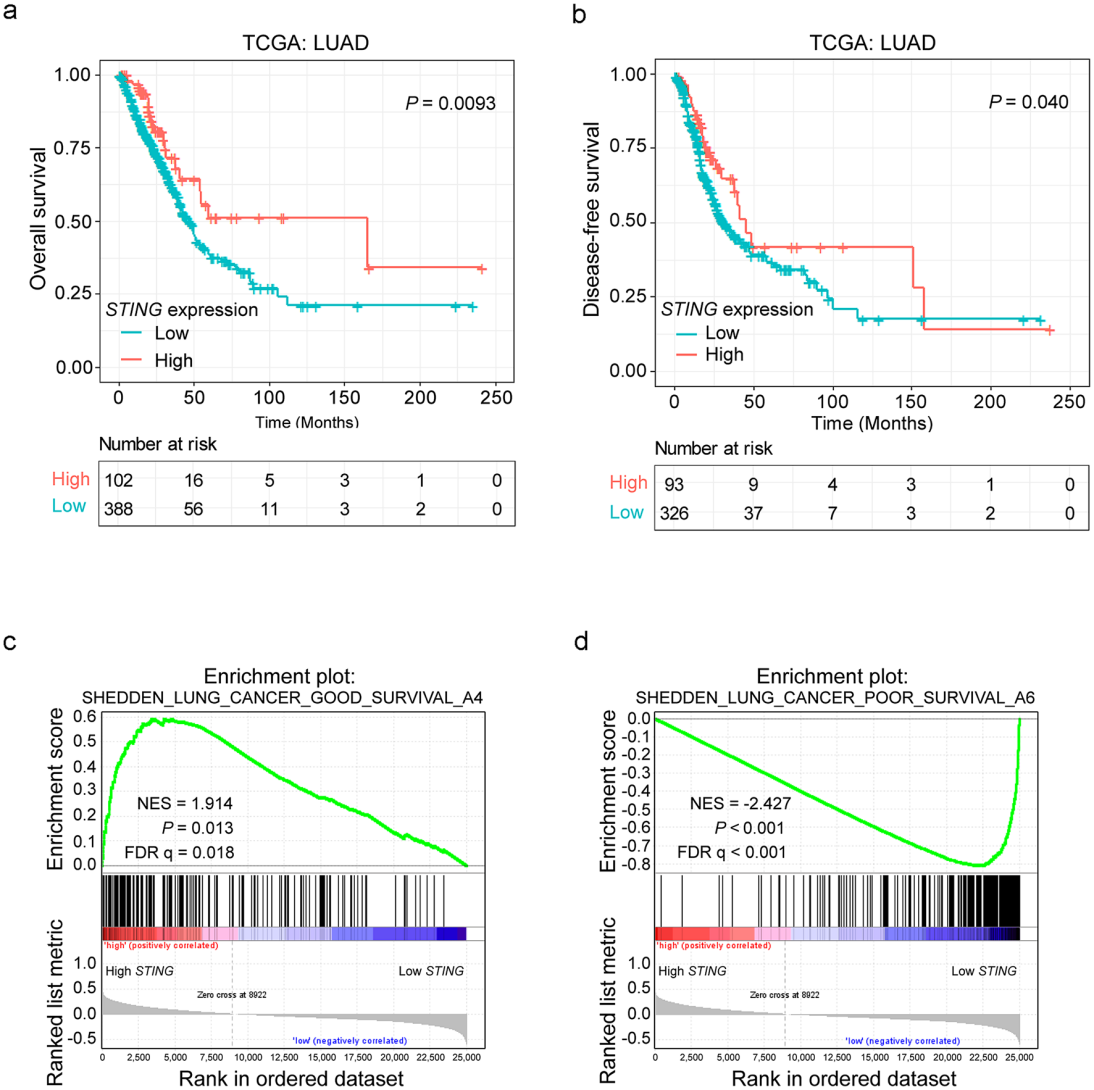
Impact of *STING* expression on LUAD patient outcomes in the TCGA dataset. (**a, b**) Kaplan–Meier survival analysis was performed to assess the association of *STING* expression with OS (**a**) and DFS (**b**) in the TCGA LUAD patients. (**c, d**) GSEA plots of enrichment of SHEDDEN_LUNG_CANCER_GOOD_SURVIVAL_A4 signatures (**c**), and SHEDDEN_LUNG_CANCER_POOR_SURVIVAL_A6 signatures (**d**) in *STING*^high^ versus *STING*^low^ tumors in the TCGA LUAD dataset.

We further searched The Cancer Genome Atlas (TCGA) LUAD dataset, an independent cohort, to validate the correlation between *STING* expression and survival probability. We first applied the “surv_cutpoint” function of the “survminer” R package to determine the optimal cutoff value of *STING* expression. We split the LUAD patient cohort into two groups: 102 of 490 patients with a *STING* expression value > 43.31 and 388 of 490 patients with a *STING* expression value ≤ 43.31 (Supplementary Fig. S1). We then performed Kaplan–Meier analysis to evaluate the association between *STING* expression and the prognosis of LUAD patients. Consistent with the observations above, LUAD patients with decreased *STING* expression had shorter OS (*P* = 0.0093, Fig. 3a**)** and DFS (*P* = 0.040, Fig. 3b). Intriguingly, by gene set enrichment analysis (GSEA) analyses, we found that the genes associated with favorable survival outcomes of lung cancer patients were enriched in LUAD patients with high *STING* expression [Normalized enrichment score (NES) = 1.914, *P* = 0.013, false discovery rate (FDR) q = 0.018, Fig. 3c and Supplementary Table S1]. In contrast, the genes associated with poor survival outcomes of lung cancer patients were enriched in LUAD patients with low *STING* expression (NES = −2.427, *P* < 0.001, FDR q < 0.001, Fig. 3d and Supplementary Table S2). Besides, Gene Ontology (GO) analysis revealed that the genes that positively correlated with *STING* expression in TCGA LUAD (R ≥ 0.3, Supplementary Table S3) were associated with various biological processes including inflammatory response, immune response, T cell activation, and antigen processing and presentation (Supplementary Fig. S2a), whereas the genes that negatively correlated with *STING* expression (R ≤ −0.3, Supplementary Table S4) were mainly enriched in cell cycle, cell division, DNA replication, and DNA repair (Supplementary Fig. S2b). These data support the role of *STING* as a predictive indicator for the prognosis of LUAD patients.

Next, we evaluated the association between *STING* expression and clinicopathological features in TCGA LUAD patients. Chi-square analysis demonstrated that *STING* expression was associated with age (*P* = 0.017, Supplementary Table S5). Intriguingly, patients with low *STING* expression tended to show a higher frequency of lymph node metastasis, although statistical significance was not reached (*P* = 0.090, Supplementary Table S5). In support of this, we found that the *STING* expression was inversely associated with metastasis signatures (NES = −2.167, *P* < 0.001, FDR q < 0.001, Supplementary Fig. S3a and Supplementary Table S6; NES = 1.757, *P* = 0.009, FDR q = 0.066, Supplementary Fig. S3b and Supplementary Table S7).

To further investigate whether *STING* could serve as an independent prognostic predictor for LUAD patients, we conducted univariate and multivariate analyses based on Cox regression analysis of the TCGA LUAD patients. Distant metastasis was excluded from the univariate and multivariate analysis, for the distant metastasis status of a high percentage of patients was unknown (Supplementary Table S5). As shown in Supplementary Table S8, after univariate analysis, 4 factors, including tumor depth (HR = 2.297, 95% CI: 1.559–3.385, *P* < 0.001), lymph node metastasis (HR = 2.657, 95% CI: 1.969–3.587, *P* < 0.001), tumor stage (HR = 2.628, 95% CI: 1.919–3.597, *P* < 0.001), and *STING* expression (HR = 0.562, 95% CI: 0.362–0.873, *P* = 0.010), were identified as risk factors for OS of LUAD patients. These 4 factors were included in the multivariate analysis. Multivariate analysis further demonstrated that tumor depth (HR = 1.815, 95% CI: 1.181–2.789, *P* = 0.007), lymph node metastasis (HR = 2.203, 95% CI: 1.543–3.146, *P* < 0.001), and *STING* expression (HR = 0.636, 95% CI: 0.408–0.991, *P* = 0.045) remained as the independent prognostic factors for OS of LUAD patients among the variables examined (Supplementary Table S8). In regarding to DFS, as shown in Supplementary Table S9, tumor depth (HR = 2.286, 95% CI: 1.469–3.558, *P* < 0.001) and lymph node metastasis (HR = 1.862, 95% CI: 1.317–2.631, *P* < 0.001) could also serve as the independent prognostic factors, but S*TING* expression failed to be of independent prognostic significance for the DFS of LUAD patients among the variables examined (HR = 0.693, 95% CI: 0.467–1.028, *P* = 0.069).

### Elevated methylation of STING may be helpful for the diagnosis of LUAD

**Figure 4:**
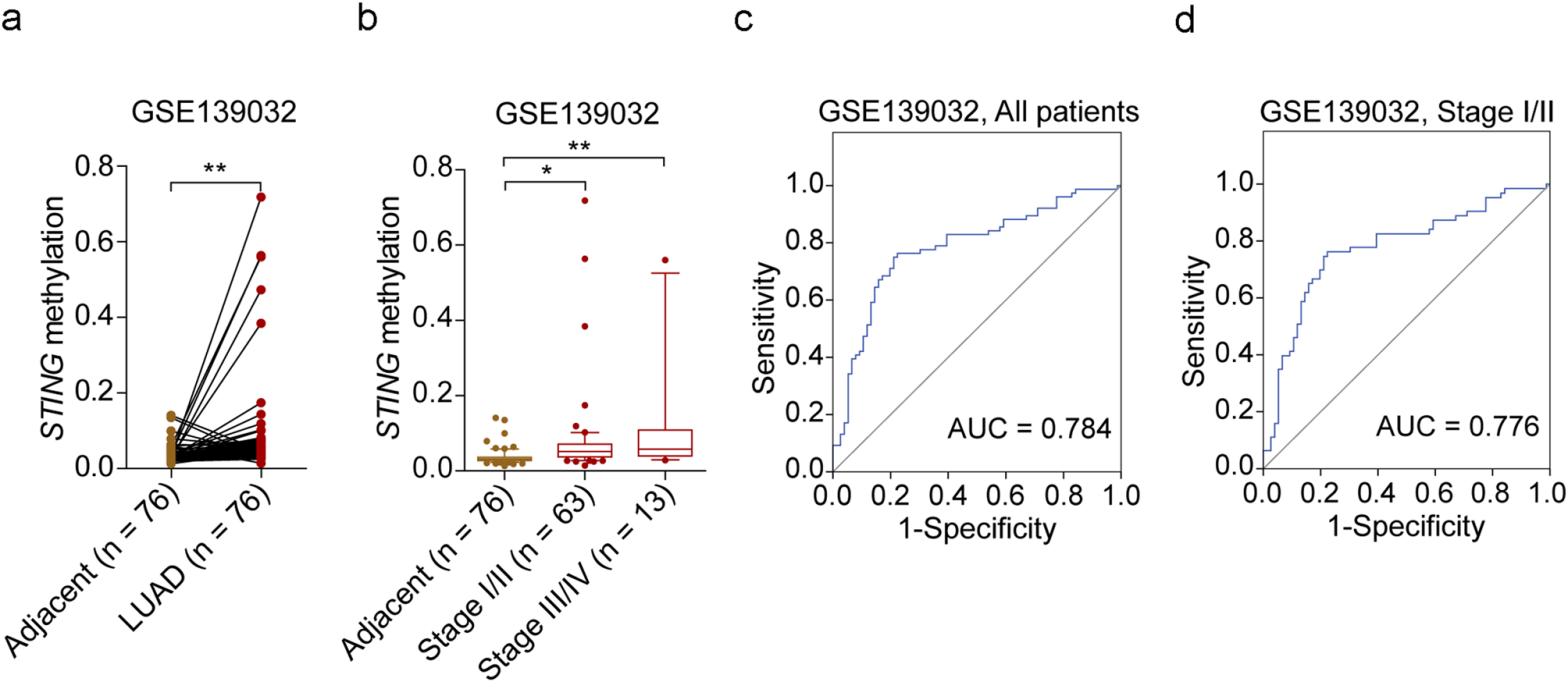
The discriminative potential of *STING* methylation in LUAD. (**a, b**) *STING* methylation status in all LUAD patients (**a**) and early-stage (Stage I/II) and advanced-stage (Stage III/IV) patients (**b**) was investigated in GSE139032. (**c**, **d**) ROC analysis of *STING* methylation in all patients (**c**) and early-stage patients (**d**) in GSE139032. Box-and-whisker plot (**b**) shows the median and 10–90th percentile of the *STING* methylation values. ^*^P < 0.05, ^**^P < 0.01.

Since *STING* methylation has been observed in TCGA LUAD patients^19^, and there was a negative correlation between *STING* methylation and its mRNA expression in the TCGA LUAD cohort (R = −0.6, *P* < 0.001, Supplementary Fig. 4). We further validated the DNA methylation status of *STING* in an independent LUAD patient cohort and found *STING* methylation levels were elevated in LUAD patients in the GSE139032 dataset (*P* < 0.01, Fig. 4a). Remarkably, the methylation levels of *STING* were found to be elevated even in early-stage (Stage I/II) LUAD patients (*P* < 0.05, Fig. 4b). Receiver operating characteristic (ROC) curve analysis was further performed to evaluate the discriminative potential of *STING* methylation. The results showed that *STING* methylation had an area under the curve (AUC) of 0.784 (sensitivity = 76.32%, specificity = 77.63%) (Fig. 4c), which distinguished LUAD tissues from adjacent non-tumor lung tissues. Of note, *STING* methylation also showed high discriminative accuracy in distinguishing early-stage LUAD tissues from adjacent non-tumor lung tissues, with an AUC of 0.776 (sensitivity = 76.19%, specificity = 77.63%) (Fig. 4d). These data suggest that aberrant *STING* methylation has the potential for the diagnosis of LUAD, even those at the early-stage.

### Aberrant STING methylation is associated with adverse outcomes in LUAD patients

**Figure 5:**
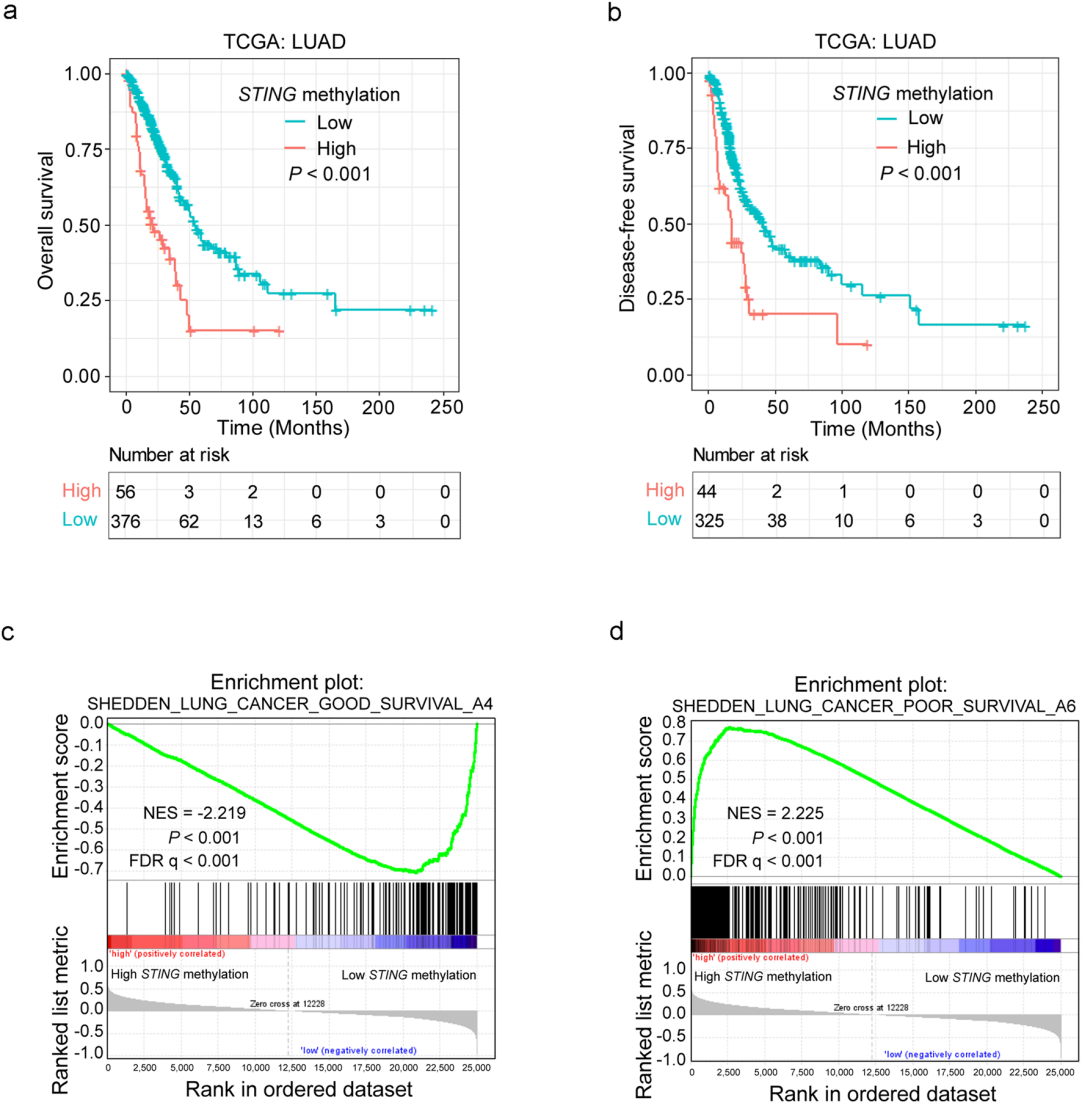
Deregulation of *STING* methylation is associated with adverse prognosis in LUAD. (**a, b**) Kaplan–Meier survival analysis was performed to assess the association of *STING* methylation with OS (**a**) and DFS (**b**) in TCGA LUAD patients. (**c, d**) GSEA plots of enrichment of SHEDDEN_LUNG_CANCER_GOOD_SURVIVAL_A4 signatures (**c**), and SHEDDEN_LUNG_CANCER_POOR_SURVIVAL_A6 signatures (**d**) in *STING* methylation^high^ versus *STING* methylation^low^ tumors in the TCGA LUAD dataset.

To further explore the clinical significance of *STING* methylation in LUAD patients, we searched the TCGA LUAD dataset to investigate whether *STING* methylation is associated with the prognosis of LUAD patients. The “surv_cutpoint” function of the “survminer” R package was used again to determine the optimal cutoff value of *STING* methylation. Then the LUAD patient cohort (with methylation data and matched clinical data) was divided into two groups: 56 of 432 patients with a *STING* methylation value > 0.54, and 376 of 432 patients with a *STING* methylation value ≤ 0.54 (Supplementary Fig. S5). Kaplan–Meier analysis showed that LUAD patients with high *STING* methylation had shorter OS (*P* < 0.001, Fig. 5a) and DFS (*P* < 0.001, Fig. 5b**)**. In support of the results obtained by the *STING* expression profile analysis, GSEA analyses revealed that the genes associated with favorable survival outcomes of lung cancer patients were enriched in LUAD patients with low *STING* methylation (NES = −2.219, *P* < 0.001, FDR q < 0.001, Fig. 5c). In contrast, the genes associated with poor survival outcomes of lung cancer patients were enriched in LUAD patients with high *STING* methylation (NES = 2.225, *P* < 0.001, FDR q < 0.001, Fig. 5d). These data indicate that *STING* methylation is a poor prognostic factor for the clinical outcome of LUAD patients.

Next, we assessed the associations between *STING* methylation and clinical features in the TCGA LUAD patients. As shown in Supplementary Table S10, there was a significant correlation between *STING* methylation and age (*P* = 0.002), gender (*P* = 0.001), stage (*P* = 0.007), and distant metastasis (*P* = 0.039), respectively. In support, we found that *STING* methylation was positively associated with metastasis signatures (NES = 2.007, *P* < 0.001, FDR q = 0.007, Supplementary Fig. S6a; NES = −2.231, *P* < 0.001, FDR q = 0.002, Supplementary Fig. S6b).

Again, we performed univariate and multivariate analyses to investigate further whether *STING* methylation could serve as an independent prognostic predictor for TCGA LUAD patients. Distant metastasis was excluded from the univariate and multivariate analysis, for the distant metastasis status of a high percentage of patients was unknown (Supplementary Table S10). As shown in Table 1, in the univariate analysis, 4 factors, including tumor depth (HR = 1.997, 95% CI: 1.296–3.079, *P* = 0.002), lymph node metastasis (HR = 2.550, 95% CI: 1.854–3.509, *P* < 0.001), stage (HR = 2.534, 95% CI: 1.809–3.551, *P* < 0.001), and *STING* methylation (HR = 2.720, 95% CI: 1.862–3.975, *P* < 0.001) were associated with an increased risk of poor OS of LUAD patients. These 4 factors were included in the multivariate analysis. Multivariate analysis further demonstrated that tumor depth (HR = 1.903, 95% CI: 1.184–3.056, *P* = 0.008) and lymph node metastasis (HR = 2.310, 95% CI: 1.585–3.365, *P* < 0.001), and also *STING* methylation (HR = 2.927, 95% CI: 1.984–4.321, *P* < 0.001) remained the independent prognostic factors for OS of LUAD patients among the variables examined (Table 1). Similarly, as shown in Table 2, tumor depth (HR = 2.615, 95% CI: 1.624–4.210, *P* < 0.001), lymph node metastasis (HR = 1.900, 95% CI: 1.313–2.749, *P* = 0.001) and *STING* methylation (HR = 2.619, 95% CI: 1.742–3.938, *P* < 0.001) could also serve as the independent prognostic factors for DFS of LUAD patients among the variables examined. Since the univariate analysis revealed that tumor depth, lymph node metastasis, stage, and *STING* methylation were all associated with the survival of LUAD patients, we attempted to develop a more accurate predictive model for outcomes of LUAD patients using these 4 factors. Results showed that the combination of *STING* methylation and tumor depth/lymph node metastasis/stage showed an increased prognostic accuracy (AUC = 0.683) for OS of LUAD patients than either tumor depth/lymph node metastasis/stage alone (AUC = 0.669, *P* = 0.321) though not statistically significant, or *STING* methylation alone (AUC = 0.574, *P* < 0.001) (Supplementary Fig. 7a) for OS of LUAD patients. Similarly, as shown in Supplementary Fig. 7b, combining *STING* methylation with tumor depth/lymph node metastasis/stage resulted in an increased AUC for predicting DFS of LUAD patients than the latter alone (*P* = 0.277) though not statistically significant, or *STING* methylation alone (*P* = 0.001). These results suggest that *STING* methylation can be used as an independent prognostic indicator and has the potential to improve the accuracy of clinical staging systems in predicting outcomes in LUAD patients.

**Table 1:** Univariate and multivariate analysis of the prognostic value of clinical factors and *STING* methylation regarding OS in TCGA LUAD patients.

Variables	Univariate analysis	*P*-value	Multivariate analysis	*P*-value
	HR (95% CI)		HR (95% CI)	
**Age**				
> 65 *vs.* ≤ 65	1.152 (0.838–1.584)	0.383	–	–
**Gender**				
Male *vs.* Female	1.071 (0.781–1.468)	0.670	–	–
**Tumor depth**				
T_3_-T_4_ *vs*. T_1_-T_2_	1.997 (1.296–3.079)	0.002	1.903 (1.184–3.056)	0.008
**Lymph node metastasis**				
N1-N3 *vs*. N0	2.550 (1.854–3.509)	< 0.001	2.310 (1.585–3.365)	< 0.001
**Stage**				
III-IV *vs*. I-II	2.534 (1.809–3.551)	< 0.001	1.226 (0.796–1.888)	0.355
***STING* methylation**				
High *vs*. Low	2.720 (1.862–3.975)	< 0.001	2.927 (1.984–4.321)	< 0.001

**Table 2:** Univariate and multivariate analysis of the prognostic value of clinical factors and *STING* methylation regarding DFS in TCGA LUAD patients.

Variables	Univariate analysis	*P*-value	Multivariate analysis	*P*-value
	HR (95% CI)		HR (95% CI)	
**Age**				
> 65 *vs.* ≤ 65	1.358 (0.988–1.866)	0.059	–	–
**Gender**				
Male *vs.* Female	1.034 (0.757–1.411)	0.834	–	–
**Tumor depth**				
T_3_-T_4_ *vs*. T_1_-T_2_	2.186 (1.411–3.386)	< 0.001	2.615 (1.624–4.210)	< 0.001
**Lymph node metastasis**				
N1-N3 *vs*. N0	1.850 (1.345–2.545)	< 0.001	1.900 (1.313–2.749)	0.001
**Stage**				
III-IV *vs*. I-II	1.832 (1.257–2.670)	0.002	0.999 (0.632–1.578)	0.996
***STING* methylation**				
High *vs*. Low	2.319 (1.556–3.455)	< 0.001	2.619 (1.742–3.938)	< 0.001

### Construction of prognostic nomogram for predicting survival based on STING methylation and clinicopathologic features

**Figure 6:**
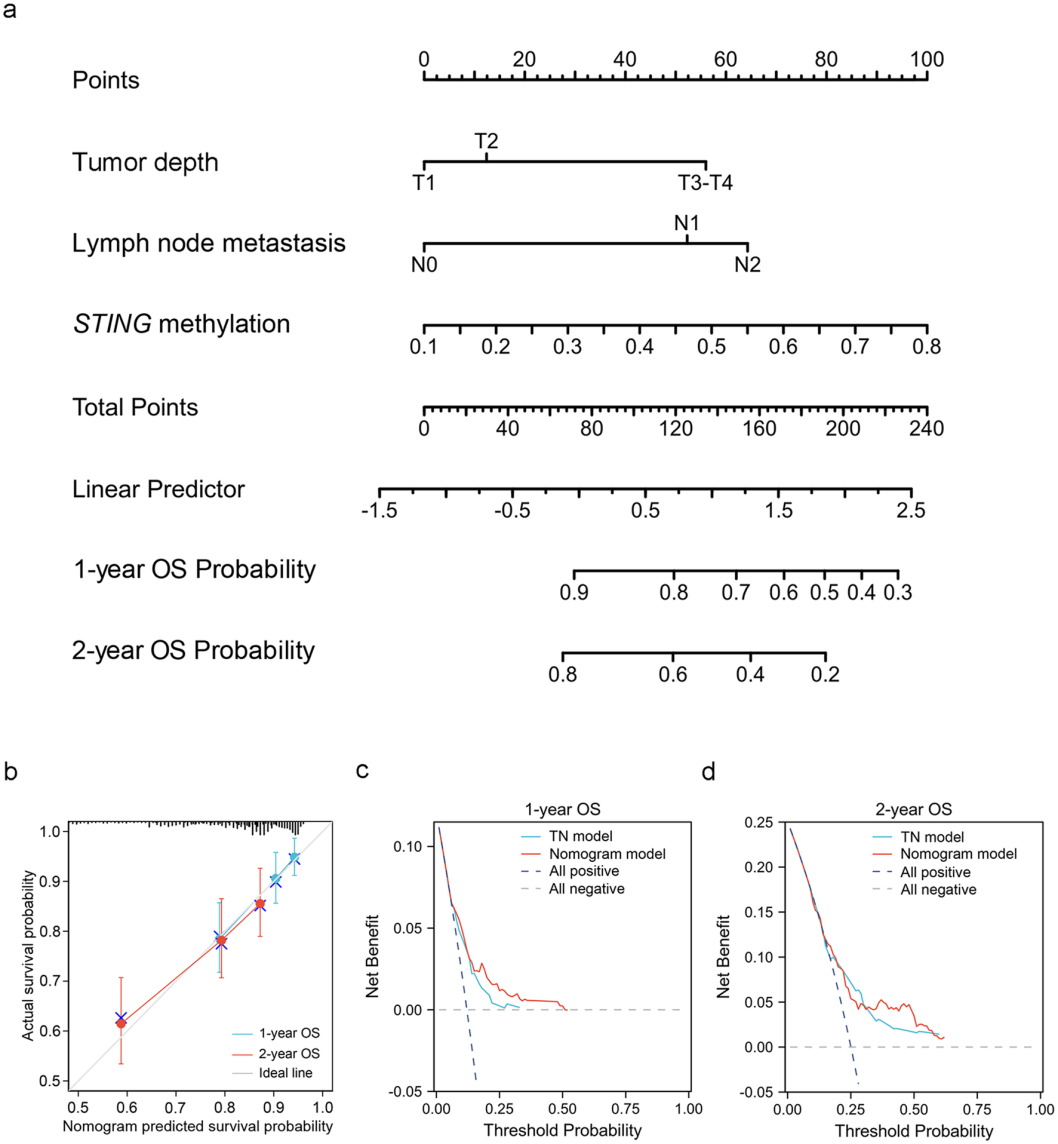
Construction and validation of the nomogram for 1- and 2-year OS of LUAD patients. (**a**) Construction of the prognostic nomogram for predicting the probability of 1- and 2-year OS in LUAD patients. The top straight line represents the points for each variable, and the total points were calculated by adding the points from each variable. A vertical line is drawn from the total point axis to the outcome axis to predict the probability of patients' survival. (**b**) Calibration plots were generated to validate agreement between the predicted and actual probability of 1- and 2-year OS of LUAD patients. (**c**, **d**) The decision curves for the nomogram predicting 1-year (**c**) and 2-year OS of LUAD patients (**d**). The x-axis depicts the probability thresholds, while the y-axis depicts the net benefit. The all positive plot assumes that all patients reached the endpoint, whereas the all negative plot assumes that none of the patients reached the endpoint. T, tumor depth; N, lymph node metastasis.

Based on the above results, we attempted to establish a nomogram for predicting the OS of LUAD patients. Since the above results showed that tumor depth, lymph node metastasis, and *STING* methylation could serve as independent prognostic indicators of OS of the TCGA LUAD patients, we built a prognostic nomogram based on these variables (Since there was only one case with N3 status, this case was excluded from this analysis) for predicting the probability of 1- and 2-year OS (Fig. 6a). Next, we created calibration curves to ascertain the concordance between the nomogram-predicted survival probability and the actual survival probability. The calibration curves revealed a good agreement between the nomogram-predicted survival probability and the actual survival probability (Fig. 6b). The decision curve analysis showed that the clinical net benefit of the nomogram model exceeded the model based on tumor depth and lymph node metastasis (Fig. 6c,d). These data suggest the favorable performance of the constructed nomogram survival model.

## Discussion

In this study, we provided evidence that the deregulation of *STING* expression and methylation was associated with LUAD clinical features, OS and DFS. More importantly, *STING* methylation can serve as an independent prognostic factor for both OS and DFS of LUAD patients. Furthermore, we built a nomogram for survival prediction based on the independent prognostic variables identified here, which include *STING* methylation, tumor depth, and lymph node metastasis. The nomogram exhibited a favorable predictive accuracy for predicting the probability of 1- and 2-year OS.

The cGAS-STING pathway, which was initially found to function in pathogen detection, has recently been demonstrated to be involved in the inhibition of cancer initiation and progression^4–7^. It has been reported that the *STING* mRNA level is downregulated in lung cancer tissues^19^. In this study, we systematically examined both the mRNA and protein levels of STING, in lung cancers, from multiple databases. In line with the previous report, our study showed that both mRNA and protein levels of STING in lung cancers were decreased in lung cancer cell lines and tissues. Of note, we showed that *STING* expression exhibited the potential to serve as a prognostic indicator for predicting survival probability in LUAD patients but not LUSC patients. Consistently, *STING* expression was positively associated with those genes that predict good survival, and negatively associated with those genes that predict poor survival in lung cancer. In support of our analysis, downregulation of *STING* mRNA expression has been associated with a poor prognosis in stage I LUAD patients^32^. Recently, immunohistochemistry analysis revealed that STING protein levels decrease in NSCLC tissues as tumor stage increases and that low STING protein levels predict a poor prognosis^33^. Furthermore, downregulation of STING can predict adverse outcomes for gastric cancer, hepatocellular carcinoma, breast cancer, and colorectal cancer^11,15,16^. Besides, *STING* was found to be positively correlated with the infiltration of various immune cells in diverse types of cancer, including LUAD^16,19^, suggesting LUAD patients with high STING expression may benefit from immune cell infiltration. However, high STING expression in tumor-infiltrating lymphocytes is significantly related to reduced OS and DFS of esophageal squamous cell carcinoma patients^34^. These data suggest that STING exerts different biological functions depending on the context.

Several reports have shown that *STING* is methylated, and the expression of *STING* is associated with DNA methylation status in a pan-cancer analysis^18,19^. Of note, DNA methyltransferase 1 (DNMT1) is a mediator of STING repression^35^ and occupies the *STING* promoter region by interacting with NEAT1 to inhibit *STING* expression in lung cancer^12^. Recently, a report demonstrated that the demethylating agent 5'AZADC is sufficient to induce the expression of STING in NSCLC cell lines^33^. Consistent with these reports, we showed that *STING* was methylated in another LUAD patient cohort, and a strong negative correlation between STING expression and methylation was observed in LUAD tissues. Contrary to the expression of *STING*, *STING* methylation predicted poor outcomes for LUAD patients. Of note *STING* methylation was upregulated in even early-stage LUAD patients and, as such, has the potential for discriminating early-stage LUAD tissues from adjacent non-tumor lung tissues. Aberrant DNA methylation has been observed frequently in a variety of cancers, including lung cancer, and has diverse implications in tumorigenesis and diagnosis^36–38^. In lung cancer patients, frequent methylation of cancer-related genes has already been observed not only in carcinoma tissues but also in various biological samples including bronchial brushing samples, sputum samples, and blood samples^38^. For instance, one study found that SHOX2 gene methylation in plasma samples has a sensitivity of 60% and a specificity of 90% in distinguishing between the lung cancer and control group^39^. In another study, a methylation panel of six genes (CDO1, HOXA9, AJAP1, PTGDR, UNCX, and MARCH11) in serum samples was revealed to correctly distinguish between stage IA NSCLC and control subjects with a sensitivity of 72.1% and a specificity 71.4%^40^. In addition, a three-gene methylation model (the combination of CDO1, TAC1, and SOX17 for sputum samples; the combination of TAC1, HOXA7, and SOX17 for plasma samples) was reported to discriminate stages I-II NSCLC from control subjects with a sensitivity of 93% and a specificity of 62% in plasma samples and a sensitivity 98% and a specificity of 71% in sputum samples^41^. While our study suggests that *STING* methylation had the potential to be a tissue biomarker for the diagnosis of LUAD, whether it can be detected in biofluids including bronchial brushing samples, sputum, and blood as a noninvasive biomarker for LUAD deserves further investigation.

Intriguingly, *STING* methylation can serve as an independent prognostic predictor of both OS and DFS for LUAD patients. Importantly, we showed that *STING* methylation was positively associated with those genes that predict poor survival, and negatively associated with those genes that predict good survival in lung cancer. We further developed a nomogram, for predicting OS, based on independent prognostic indicators, including *STING* methylation, tumor depth, and lymph node metastasis. The predictive efficacy of the model was examined by calibration curves. The models exhibited favorable accuracy for 1- and 2-year OS prediction. Further prospective studies are required to validate the model.

The role of cGAS-STING pathway in cancer is quite complicated. Usually, activation of the cGAS-STING pathway is sufficient to recruit effector T cells into the tumor microenvironment and eliminate the tumor cells^8–10^. In lung cancer, there are multiple lines of evidence suggesting that cGAS-STING signaling functions as a tumor suppressor. A recent study found that DNA damage response (DDR) inhibitors are sufficient to induce an anti-tumor immune response in small cell lung cancer, which is mediated by the STING-TANK binding kinase 1 (TBK1)-IFN regulatory factor 3 (IRF3) pathway^13^. Another study discovered that the natural product rocaglamide specifically damages mtDNA and promotes its cytoplasmic release, which stimulates the activation of cGAS-STING signaling, resulting in increased natural killer (NK) cell infiltration and tumor growth suppression in NSCLC^42^. Moreover, cGAS-STING signaling was demonstrated to be activated by DNA damage caused by ribonucleotide reductase regulatory subunit M2 (RRM2), which then suppresses malignant phenotype and improves radiosensitivity in LUAD^43^. Furthermore, a study showed that lung tumors with MET amplification can develop resistance to immune checkpoint blockade treatment through downregulation of STING expression^44^. In addition, Sex-determining region Y-related high-mobility group box 2 (SOX2) was found to occupy the cGAS promoter and repress its transcription, then dampen cGAS/STING signaling and ultimately inhibit ionizing radiation-induced anti-tumor immune responses in NSCLC^45^. More importantly, the activation of the cGAS-STING pathway by dimeric amidobenzimidazole (diABZI), a STING agonist, has recently been shown to sensitize NSCLC cells to irradiation by promoting apoptosis^46^. And targeting STING with DMXAA, a STING agonist, is sufficient to improve innate and adaptive immune signaling in Kelch-like ECH-associated protein 1 (KEAP1)-mutant NSCLC tumors, which are frequently resistant to immunotherapy^47^. These studies suggest that STING exhibits tumor-suppressive effects in lung cancer. However, emerging data suggest that activation of the cGAS-STING pathway can also contribute to tumorigenesis by activation of immunoregulatory mechanisms^8,48–50^. For example, a study showed that STING can induce indoleamine 2,3 dioxygenase (IDO) in the tumor microenvironment to suppress tumor-infiltrating lymphocytes (TILs) infiltration and then promotes the growth of Lewis lung carcinoma (LLC)^49^. Furthermore, using cyclic diadenyl monophosphate (CDA) to activate STING in the LLC mouse model not only elicits potent antitumor responses but also stimulates a rapid increase of immunoregulatory pathways involving PD-1, IDO, and COX2 in the tumor microenvironment, which then attenuates antitumor responses. Blocking each pathway individually improves CDA-induced antitumor immunity^50^. In this study, *STING* expression was found to be inversely correlated with lymph node metastasis, and its methylation was found to be positively correlated with distant metastasis. Consistently, using GSEA, we discovered that *STING* expression was negatively associated with, whereas *STING* methylation was positively associated with metastasis gene signatures. Go analysis also suggested a critical role of STING in LUAD. In support, downregulation of STING was found to be positively associated with tumor invasion, lymph node metastasis, and lymphovascular invasion in various cancer types^11,15,16^. In addition, STING has been implicated in the inhibition of migration and invasion of lung cancer cells^12^. Knockdown of STING promotes migration and invasion of gastric cancer cells^11^. Thus, targeting STING by STING agonists may suppress the metastasis of lung cancer, which is worthy of further investigation.

Nonetheless, whether the expression and methylation of *STING* can be used as potential markers for LUAD needs to be verified further in a series of independent cohorts and validate their clinical significance. Moreover, the biological function and underlying mechanisms of STING in LUAD deserve to be investigated by performing in vitro and in vivo studies.

## Conclusion

In the present study, we investigated the prognostic value of *STING* expression and its methylation in LUAD patients. We showed that both mRNA expression and methylation of *STING* can predict the outcome of LUAD patients. *STING* methylation has the potential to be an independent prognostic indicator for LUAD patient outcomes. We also established a nomogram that exhibits favorable predictive accuracy for 1- and 2-year survival for OS. Therefore, our findings support that both mRNA expression and methylation of *STING* have a potential prognostic role in LUAD.

## Materials and methods

### Ethics statement

The data used in the study was obtained from public resources, and hence the study was exempt from a local ethics committee approval. All methods were performed in accordance with the relevant guidelines and regulations.

### Expression and methylation analysis

The expression of *STING* across various human tissues was investigated, using the data from GTEx (https://www.gtexportal.org/)^26^, with the access provided by the Human Protein Atlas (https://www.proteinatlas.org/)^27^. Expression data of *STING* in a variety of lung cancer cell lines were downloaded from the MERAV database (http://merav.wi.mit.edu)^28^. The immunohistochemical intensity of STING in LUAD, LUSC, and normal lung tissues was investigated in the Human Protein Atlas. Protein expression of STING was analyzed in Clinical Proteomic Tumor Analysis Consortium (CPTAC) via the access provided by the UALCAN database (http://ualcan.path.uab.edu/analysis-prot.html)^29,30^. We obtained the GSE139032 dataset from Gene Expression Omnibus (GEO; https://www.ncbi.nlm.nih.gov/geo)^51^, and used the probe cg16983159 to detect the DNA methylation of *STING* in LUAD and paired adjacent non-tumor lung tissues of GSE139032 (n = 77, the methylation value of *STING* in one of the 77 LUAD samples was not available)^52^. We used STING alias “TMEM173” to extract the expression data of STING in Human Protein Atlas (the antibody HPA038116 was used to detect STING staining), MERAV, and UALCAN databases.

### Survival analysis

Kaplan–Meier survival analysis was performed to assess the association between *STING* expression and OS of LUAD and LUSC using Kaplan–Meier Plotter (http://www.kmplot.com/analysis/index.php?p=service&cancer=lung)^31^. Probe 224929_at was used to detect *STING* in Kaplan–Meier Plotter, and the option “Auto select best cutoff” was selected to determine the cutoff point of the *STING* expression.

RNA-seq, DNA methylation, and clinical data of the LUAD patients were downloaded from TCGA ( https://cancergenome.nih.gov/) portal The NCI's Genomic Data Commons (https://gdc.cancer.gov), and the cBioPortal (http://www.cbioportal.org/)^53^. 504 out of 513 patients (504 patients with RNA-seq data and 443 patients with methylation data) had intact follow-up data. The patients with a follow-up of less than one month were excluded from the survival analysis (490 patients with RNA-seq data had OS data ≥ 1 month; 432 patients with methylation data had OS data ≥ 1 month). Among these patients, 419 patients with RNA-seq expression profiles had DFS data and 369 patients with methylation profiles had DFS data. We used the STING alias “TMEM173” to extract the expression and methylation data of *STING* (The probe cg04232128 was used to detect the methylation levels of *STING*). The optimal cutoff values of *STING* expression and methylation were determined by the “surv_cutpoint” function of the “survminer” R package^54,55^. TCGA LUAD patients were dichotomized into high and low groups according to each optimal cut-off value. We then performed the Kaplan–Meier survival analysis of mRNA expression and methylation for *STING* in the above patients, using the R package “survival”. Univariate and multivariate Cox proportional hazards models were applied to evaluate the survival data.

### Construction and assessment of the predictive nomogram survival models

Nomogram is a graphical model that predicts the occurrence of events. The clinical parameters of patients were assigned a point in the nomogram's graphic interface. A straight line represents the points (a range of 0 to 100) for each variable, while the sum of the points for each variable was quantified as the total points. Patients' survival probabilities were then examined by drawing vertical lines from the total point axis to the outcome axis. A nomogram survival model was constructed based on *STING* methylation and clinicopathologic features, to predict the probability of 1- and 2-year OS. Calibration curves were generated to evaluate the concordance between the nomogram survival model-predicted survival probability and actual survival probability. The decision curve analysis was carried out to assess the clinical usefulness of the nomogram model.

### Gene set enrichment analysis

The mRNA profiles of 490 TCGA LUAD patients with RNA-seq data and 432 TCGA LUAD patients with methylation data were dichotomized into high- and low*-STING* groups, and high- and low*-STING* methylation groups, respectively, as described above. Then the data were subjected to the GSEA software (version 2.0.13), using gene sets obtained from http://www.gsea-msigdb.org/gsea/index.jsp, as previously described^56^.

### GO analysis

The genes that positively or negatively correlated with *STING* expression (|R|≥ 0.3) in TCGA LUAD were analyzed in the cBioPortal database, and then those genes were subjected to the DAVID database (https://david.ncifcrf.gov/) to analyze their biological function.

### Statistical analysis

We performed statistical analyses using the SPSS 17.0 software (SPSS Inc., USA) or R packages. Student’s *t*-test was carried out to compare the difference between the two groups, and one-way ANOVA with post hoc intergroup comparisons was conducted to compare the difference among more than two groups. Chi-square test was used to compare categorical variables. ROC was applied to evaluate the discriminative power of various variables in LUAD patients. We applied R packages “ggplot2” and “ggpubr” to assess the correlation between *STING* methylation and its mRNA expression in LUAD tissues. All statistical results with a *P*-value < 0.05 were considered to be statistically significant.

## Supplementary Information

Supplementary Information 1.

Supplementary Information 2.

## Data Availability

The data that support the findings of this study are available from the Human Protein Atlas (https://www.proteinatlas.org), MERAV database (http://merav.wi.mit.edu), UALCAN database (http://ualcan.path.uab.edu/analysis-prot.html), GEO (https://www.ncbi.nlm.nih.gov/geo), Kaplan–Meier Plotter (http:// www.kmplot.com/analysis/index.php?p=service&cancer=lung), The NCI's Genomic Data Commons (https://gdc.cancer.gov), and cBioPortal (http://www.cbioportal.org/).

## References

[1] Ishikawa H, Barber GN (2008). STING is an endoplasmic reticulum adaptor that facilitates innate immune signalling. Nature.

[2] Kato K, Omura H, Ishitani R, Nureki O (2017). Cyclic GMP-AMP as an endogenous second messenger in innate immune signaling by cytosolic DNA. Annu. Rev. Biochem..

[3] Ma Z, Damania B (2016). The cGAS-STING defense pathway and its counteraction by viruses. Cell. Host Microbe..

[4] Ahn J, Konno H, Barber GN (2015). Diverse roles of STING-dependent signaling on the development of cancer. Oncogene.

[5] Zhu Q (2014). Cutting edge: STING mediates protection against colorectal tumorigenesis by governing the magnitude of intestinal inflammation. J. Immunol..

[6] Ohkuri T (2014). STING contributes to antiglioma immunity via triggering type I IFN signals in the tumor microenvironment. Cancer Immunol. Res..

[7] Ohkuri T (2015). Protective role of STING against gliomagenesis: Rational use of STING agonist in anti-glioma immunotherapy. Oncoimmunology.

[8] Zhou X, Jiang Z (2017). STING-mediated DNA sensing in cancer immunotherapy. Sci. China Life Sci..

[9] Woo SR (2014). STING-dependent cytosolic DNA sensing mediates innate immune recognition of immunogenic tumors. Immunity.

[10] Deng L (2014). STING-dependent cytosolic DNA sensing promotes radiation-induced type I interferon-dependent antitumor immunity in immunogenic tumors. Immunity.

[11] Song S (2017). Decreased expression of STING predicts poor prognosis in patients with gastric cancer. Sci. Rep..

[12] Ma F (2020). LncRNA NEAT1 Interacted With DNMT1 to regulate malignant phenotype of cancer cell and cytotoxic T Cell infiltration via epigenetic inhibition of p53, cGAS, and STING in lung cancer. Front Genet..

[13] Sen T (2019). Targeting DNA damage response promotes antitumor immunity through STING-mediated T-cell activation in small cell lung cancer. Cancer Discov..

[14] Della Corte, C. M. *et al.* STING pathway expression identifies NSCLC with an immune-responsive phenotype. *J. Thorac. Oncol*. **15**, 777–791 10.1016/j.jtho.2020.01.009 (2020). 10.1016/j.jtho.2020.01.009 PMC7202130 32068166

[15] Bu Y, Liu F, Jia QA, Yu SN (2016). Decreased expression of TMEM173 predicts poor prognosis in patients with hepatocellular carcinoma. PLoS ONE.

[16] Yang H (2019). STING activation reprograms tumor vasculatures and synergizes with VEGFR2 blockade. J. Clin. Invest..

[17] Luo WW, Shu HB (2018). Delicate regulation of the cGAS-MITA-mediated innate immune response. Cell. Mol. Immunol..

[18] Konno H (2018). Suppression of STING signaling through epigenetic silencing and missense mutation impedes DNA damage mediated cytokine production. Oncogene.

[19] An X (2019). An analysis of the expression and association with immune cell infiltration of the cGAS/STING pathway in pan-cancer. Mol. Ther. Nucleic Acids..

[20] Bray F (2018). Global cancer statistics 2018: GLOBOCAN estimates of incidence and mortality worldwide for 36 cancers in 185 countries. CA Cancer J. Clin..

[21] Tan WL (2016). Novel therapeutic targets on the horizon for lung cancer. Lancet Oncol..

[22] Chen Z (2014). Non-small-cell lung cancers: A heterogeneous set of diseases. Nat. Rev. Cancer..

[23] Skoulidis F, Heymach JV (2019). Co-occurring genomic alterations in non-small-cell lung cancer biology and therapy. Nat. Rev. Cancer..

[24] Chen J (2020). Genomic landscape of lung adenocarcinoma in East Asians. Nat. Genet..

[25] Filosso PL (2011). Adenosquamous lung carcinomas: A histologic subtype with poor prognosis. Lung Cancer.

[26] GTEx Consortium. The genotype-tissue expression (GTEx) project. *Nat. Genet*. **45**, 580–585 10.1038/ng.2653 (2013). 10.1038/ng.2653 PMC4010069 23715323

[27] Uhlen M (2015). Proteomics. Tissue-based map of the human proteome. Science.

[28] Shaul YD (2016). MERAV: A tool for comparing gene expression across human tissues and cell types. Nucleic Acids Res..

[29] Chen F, Chandrashekar DS, Varambally S, Creighton CJ (2019). Pan-cancer molecular subtypes revealed by mass-spectrometry-based proteomic characterization of more than 500 human cancers. Nat. Commun..

[30] Chandrashekar DS (2017). UALCAN: A portal for facilitating tumor subgroup gene expression and survival analyses. Neoplasia.

[31] Gyorffy B, Surowiak P, Budczies J, Lanczky A (2013). Online survival analysis software to assess the prognostic value of biomarkers using transcriptomic data in non-small-cell lung cancer. PLoS ONE.

[32] Raaby Gammelgaard, K. *et al.* cGAS-STING pathway expression as a prognostic tool in NSCLC. *Transl Lung Cancer Res*. **10**, 340–354 10.21037/tlcr-20-524 (2021). 10.21037/tlcr-20-524 PMC7867790 33569317

[33] Lohinai Z (2022). Loss of STING expression is prognostic in non-small cell lung cancer. J. Surg. Oncol..

[34] Wang ZC (2017). Expression of STING and MIF in tumor infiltration lymphocytes as prognostic factors in patients with ESCC. Int. J. Clin. Exp. Pathol..

[35] Kitajima S (2019). Suppression of STING associated with LKB1 loss in KRAS-driven lung cancer. Cancer Discov..

[36] Nishiyama A, Nakanishi M (2021). Navigating the DNA methylation landscape of cancer. Trends Genet..

[37] Seijo LM (2019). Biomarkers in lung cancer screening: Achievements, promises, and challenges. J. Thorac. Oncol..

[38] Liang R (2021). DNA methylation in lung cancer patients: Opening a "window of life" under precision medicine. Biomed. Pharmacother..

[39] Kneip C (2011). SHOX2 DNA methylation is a biomarker for the diagnosis of lung cancer in plasma. J. Thorac. Oncol..

[40] Ooki A (2017). A panel of novel detection and prognostic methylated DNA markers in primary non-small cell lung cancer and serum DNA. Clin. Cancer Res..

[41] Hulbert A (2017). Early detection of lung cancer using DNA promoter hypermethylation in plasma and sputum. Clin. Cancer Res..

[42] Yan X (2022). Rocaglamide promotes the infiltration and antitumor immunity of NK cells by activating cGAS-STING signaling in non-small cell lung cancer. Int. J. Biol. Sci..

[43] Jiang X (2021). RRM2 silencing suppresses malignant phenotype and enhances radiosensitivity via activating cGAS/STING signaling pathway in lung adenocarcinoma. Cell Biosci..

[44] Zhang Y (2021). MET amplification attenuates lung tumor response to immunotherapy by inhibiting STING. Cancer Discov..

[45] Gao Y (2022). LncRNA PCAT1 activates SOX2 and suppresses radioimmune responses via regulating cGAS/STING signalling in non-small cell lung cancer. Clin. Transl. Med..

[46] Xue A (2022). Increased activation of cGAS-STING pathway enhances radiosensitivity of non-small cell lung cancer cells. Thorac. Cancer..

[47] Marzio A (2022). EMSY inhibits homologous recombination repair and the interferon response, promoting lung cancer immune evasion. Cell.

[48] Ng KW, Marshall EA, Bell JC, Lam WL (2018). cGAS-STING and cancer: Dichotomous roles in tumor immunity and development. Trends Immunol..

[49] Lemos H (2016). STING promotes the growth of tumors characterized by low antigenicity via IDO activation. Cancer Res..

[50] Lemos H (2020). Overcoming resistance to STING agonist therapy to incite durable protective antitumor immunity. J. Immunother. Cancer.

[51] Clough E, Barrett T (2016). The gene expression omnibus database. Methods Mol. Biol..

[52] Enfield KSS (2019). Epithelial tumor suppressor ELF3 is a lineage-specific amplified oncogene in lung adenocarcinoma. Nat. Commun..

[53] Gao, J. *et al.* Integrative analysis of complex cancer genomics and clinical profiles using the cBioPortal. *Sci Signal*. **6**, pl1. 10.1126/scisignal.2004088 (2013). 10.1126/scisignal.2004088 PMC4160307 23550210

[54] Zhou R (2019). A robust panel based on tumour microenvironment genes for prognostic prediction and tailoring therapies in stage I-III colon cancer. EBioMedicine.

[55] Zeng D (2020). Macrophage correlates with immunophenotype and predicts anti-PD-L1 response of urothelial cancer. Theranostics.

[56] Du, L. *et al.* MTA3 Represses cancer stemness by targeting the SOX2OT/SOX2 axis. *iScience*, **22**, 353–368 10.1016/j.isci.2019.11.009 (2019). 10.1016/j.isci.2019.11.009 PMC6909183 31810000

