# Supplementary material for: Clinical significance of STING expression and methylation in lung adenocarcinoma based on bioinformatics analysis
Source: Sci Rep. 2022 Aug 17;12:13951. doi: 10.1038/s41598-022-18278-6 (PMC9385651; doi:10.1038/s41598-022-18278-6)
Supplement: Supplementary file 1 — Supplementary Information 1. [file 41598_2022_18278_MOESM1_ESM.pdf]

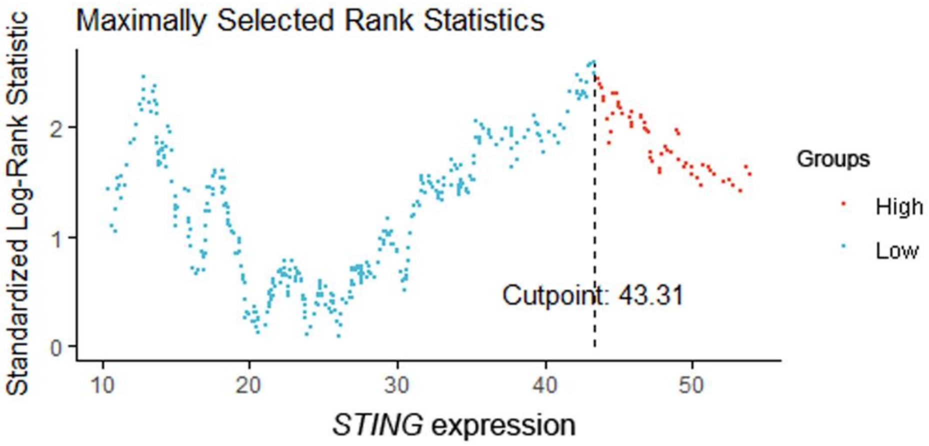

Supplementary Figure S1. Determination of the cutoff value for *STING* expression. The optimal cutoff value of *STING* expression was determined by the "surv_cutpoint" function of the "survminer" R package.

a

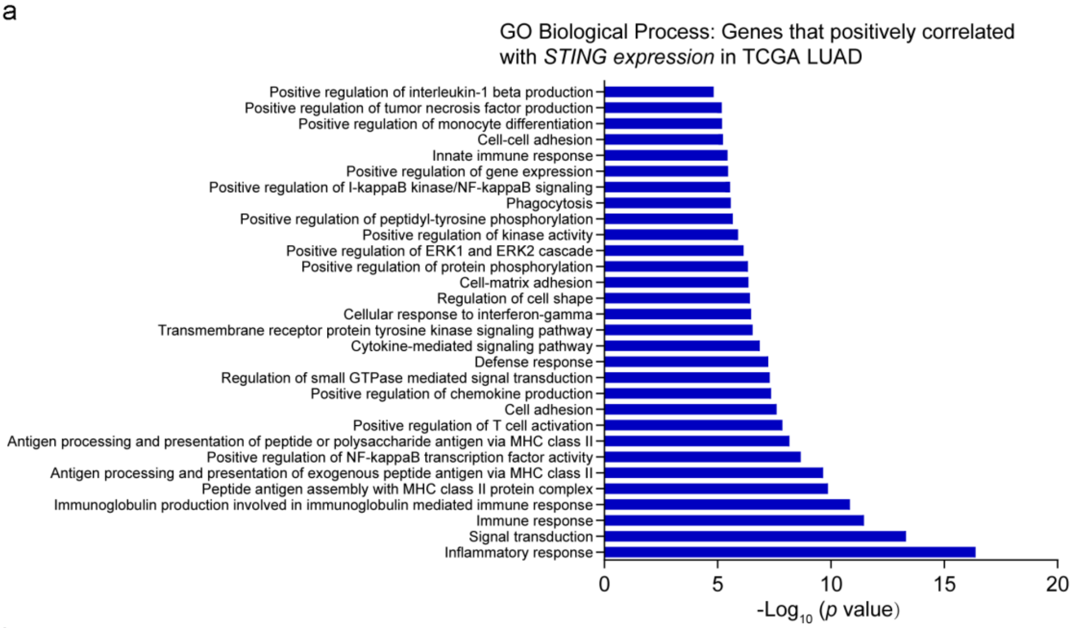

b

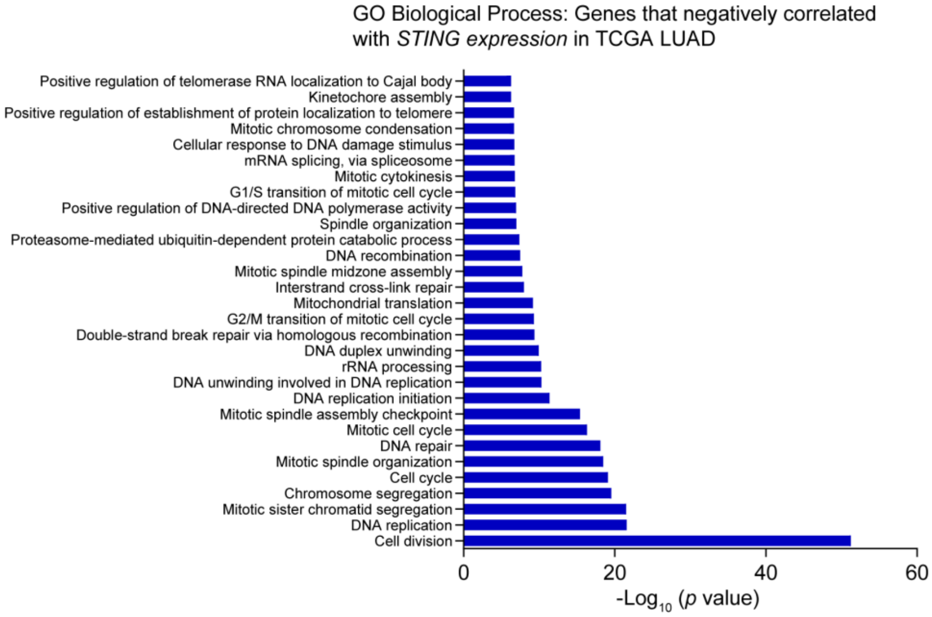

Supplementary Figure S2. STING-related genes are linked to a variety of biological processes. (**a**, **b**) GO enrichment analysis of the top 30 biological processes regulated by the genes that are positively (**a**) or negatively (**b**) correlated with *STING* in TCGA LUAD.

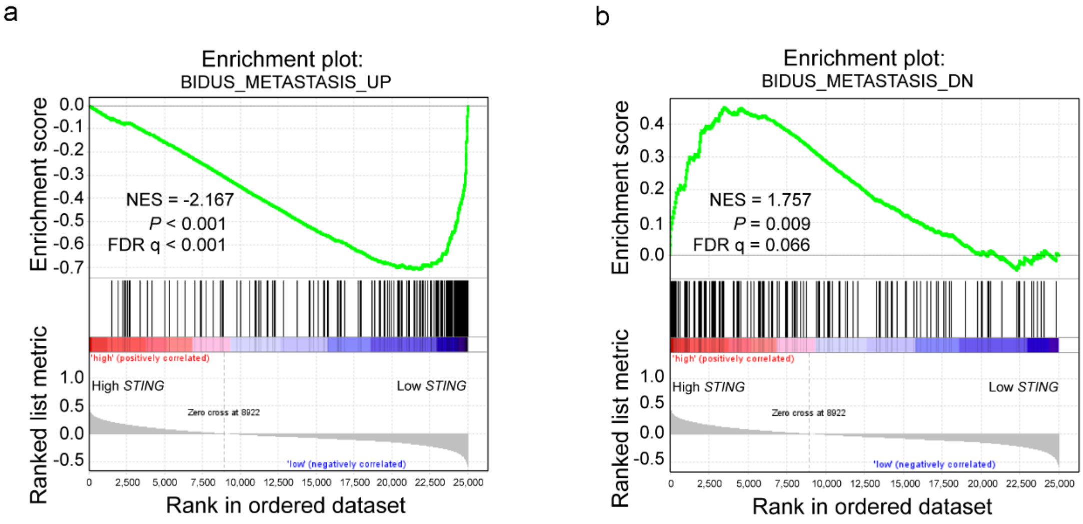

Supplementary Figure S3. *STING* expression is inversely associated with metastasis signatures. GSEA plots of enrichment of BIDUS_METASTASIS_UP signatures (**a**), and BIDUS_METASTASIS_DN signatures (**b**) in *STING*^{high} versus *STING*^{low} tumors in the TCGA LUAD dataset.

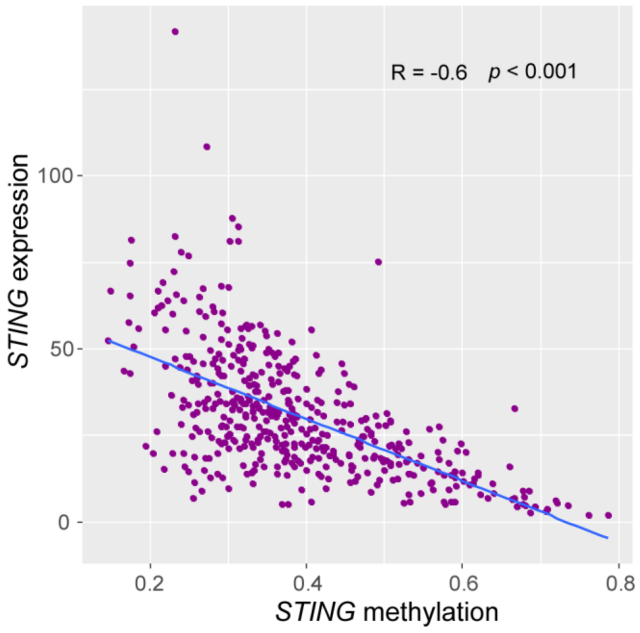

Supplementary Figure 4. Methylation of *STING* is negatively correlated with its expression. Correlation between *STING* methylation and its expression was analyzed in the TCGA LUAD dataset.

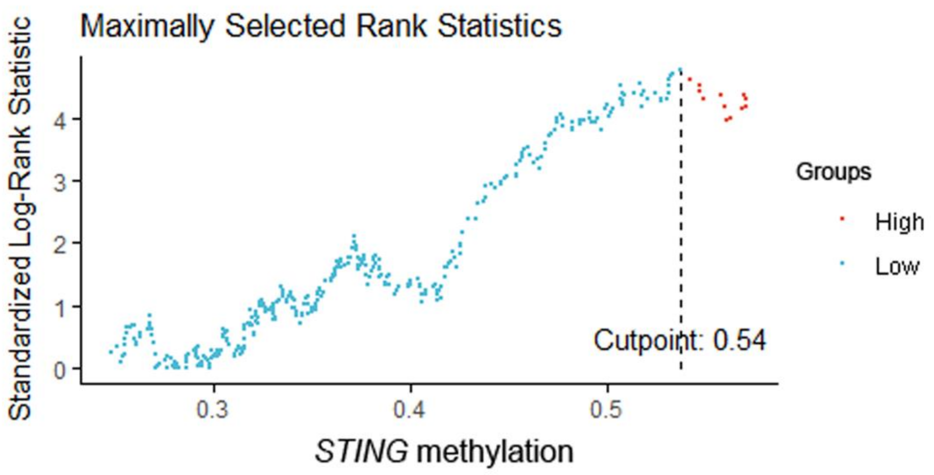

Supplementary Figure S5. Determination of the cutoff value for *STING* methylation. The optimal cutoff value of *STING* methylation was determined by the “surv_cutpoint” function of the “survminer” R package.

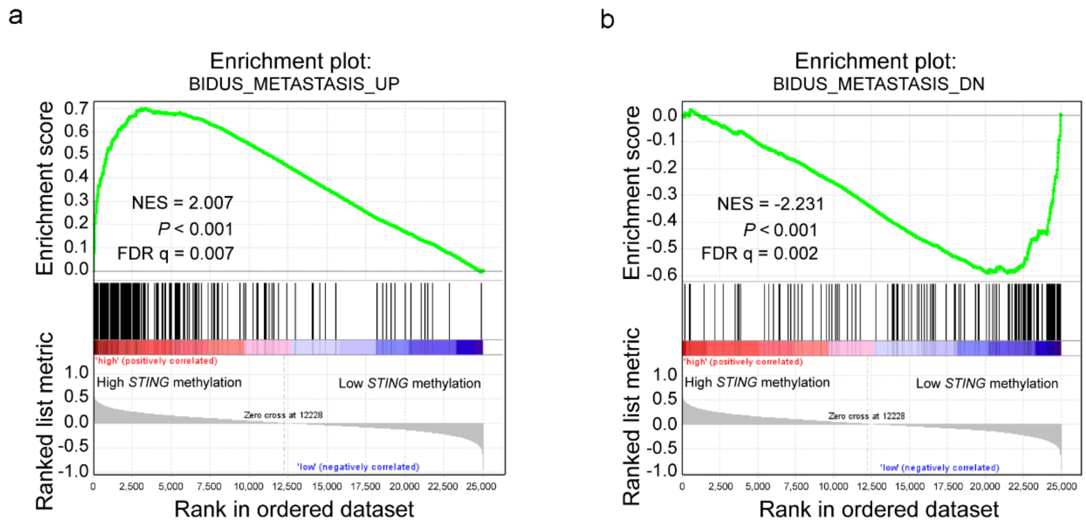

Supplementary Figure S6. *STING* methylation is positively associated with metastasis signatures. GSEA plots of enrichment of BIDUS_METASTASIS_UP signatures (**a**) and BIDUS_METASTASIS_DN signatures (**b**) in *STING* methylation^{high} versus *STING* methylation^{low} tumors in the TCGA LUAD dataset.

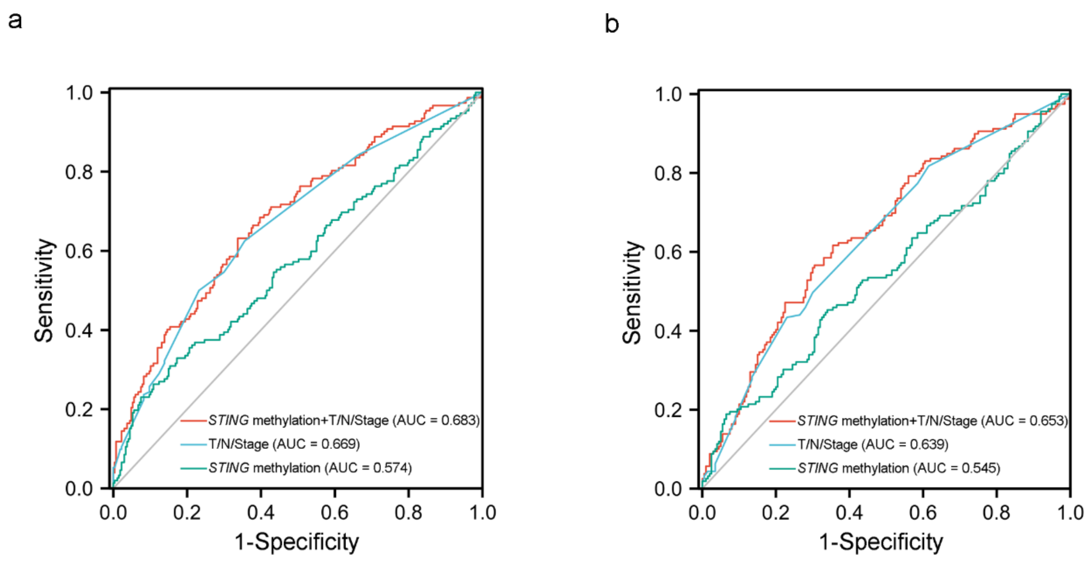

Supplementary Figure S7. ROC analysis of various variables' predictive accuracy for prognosis in LUAD patients. The predictive accuracy of *STING* methylation, T/N/Stage, and the combination of *STING* methylation and T/N/Stage for OS (**a**) and DFS (**b**) in LUAD patients was evaluated using a ROC curve. T, tumor depth; N, lymph node metastasis.
