# Supplementary material for: Clinical significance of STING expression and methylation in lung adenocarcinoma based on bioinformatics analysis
Source: Sci Rep. 2022 Aug 17;12:13951. doi: 10.1038/s41598-022-18278-6 (PMC9385651; doi:10.1038/s41598-022-18278-6)
Supplement: Supplementary file 2 — Supplementary Information 2. [file 41598_2022_18278_MOESM2_ESM.zip › Supplementary Information 2/Supplementary Table S10.docx]

**Supplementary Table S10. Associations of *STING* methylation with clinicopathological variables of TCGA LUAD patients.** ^*^Clinical information of some patients is not complete.

| Variables |  | *STING* methylation | |  |
| --- | --- | --- | --- | --- |
|  | N | Low, n (%) | High, n (%) | *P-*value |
| Patients | 432 | 376(87.4) | 56(12.6) |  |
| Age(Years)^*^ |  |  |  |  |
| >65 | 213 | 196(92.0) | 17(8.0) | 0.002 |
| ≤65 | 209 | 171(81.8) | 38(18.2) |  |
| Gender |  |  |  |  |
| Male | 204 | 166(81.4) | 38(18.6) | 0.001 |
| Female | 228 | 210(92.1) | 18(7.9) |  |
| Tumor depth^*^ |  |  |  |  |
| T1-T2 | 376 | 327(87.0) | 49(13.0) | 0.972 |
| T3-T4 | 53 | 46(86.8) | 7(13.2) |  |
| Lymph node metastasis^*^ |  |  |  |  |
| N0 | 283 | 251(88.7) | 32(11.3) | 0.096 |
| N1-N3 | 140 | 116(82.9) | 24(17.1) |  |
| Distant metastasis^*^ |  |  |  |  |
| M0 | 271 | 233(86.0) | 38(14.0) | 0.039 |
| M1 | 19 | 13(68.4) | 6(31.6) |  |
| Stage^*^ |  |  |  |  |
| I-II | 338 | 302(89.3) | 36(10.7) | 0.007 |
| Ⅲ-Ⅳ | 89 | 70(78.7) | 19(21.3) |  |
