# Supplementary material for: Clinical significance of STING expression and methylation in lung adenocarcinoma based on bioinformatics analysis
Source: Sci Rep. 2022 Aug 17;12:13951. doi: 10.1038/s41598-022-18278-6 (PMC9385651; doi:10.1038/s41598-022-18278-6)
Supplement: Supplementary file 2 — Supplementary Information 2. [file 41598_2022_18278_MOESM2_ESM.zip › Supplementary Information 2/Supplementary Table S5.docx]

**Supplementary Table S5. Associations of *STING* expression with clinicopathological variables of TCGA LUAD patients.** ^*^Clinical information of some patients is not complete.

| Variables |  | *STING* expression | |  |
| --- | --- | --- | --- | --- |
|  | N | Low, n (%) | High, n (%) | *P-*value |
| All patients | 490 | 388(79.2) | 102(20.8) |  |
| Age(Years)^*^ |  |  |  |  |
| >65 | 249 | 186(74.7) | 63(25.3) | 0.017 |
| ≤65 | 231 | 193(83.5) | 38(16.5) |  |
| Gender |  |  |  |  |
| Male | 228 | 189(82.9) | 39(17.1) | 0.059 |
| Female | 262 | 199(76.0) | 63(24.0) |  |
| Tumor depth^*^ |  |  |  |  |
| T1-T2 | 426 | 339(79.6) | 87(20.4) | 0.454 |
| T3-T4 | 61 | 46(75.4) | 15(24.6) |  |
| Lymph node metastasis^*^ |  |  |  |  |
| N0 | 317 | 243(76.7) | 74(23.3) | 0.090 |
| N1-N3 | 162 | 135(83.3) | 27(16.7) |  |
| Distant metastasis^*^ |  |  |  |  |
| M0 | 324 | 266(82.1) | 58(17.9) | 0.879 |
| M1 | 24 | 20(83.3) | 4(16.7) |  |
| Stage* |  |  |  |  |
| I-II | 378 | 295(78.0) | 83(22.0) | 0.211 |
| Ⅲ-Ⅳ | 104 | 87(83.7) | 17(16.3) |  |
