# Supplementary material for: Clinical significance of STING expression and methylation in lung adenocarcinoma based on bioinformatics analysis
Source: Sci Rep. 2022 Aug 17;12:13951. doi: 10.1038/s41598-022-18278-6 (PMC9385651; doi:10.1038/s41598-022-18278-6)
Supplement: Supplementary file 2 — Supplementary Information 2. [file 41598_2022_18278_MOESM2_ESM.zip › Supplementary Information 2/Supplementary Table S8.docx]

**Supplementary Table S8. Univariate and multivariate analysis of the prognostic value of clinical factors and *STING* expression regarding OS in TCGA LUAD patients.**

| Variables | Univariate analysis | *P*-value | Multivariate analysis | *P*-value |
| --- | --- | --- | --- | --- |
|  | HR (95% CI) |  | HR (95% CI) |  |
| Age  >65 *vs.* ≤65 | 1.197(0.888-1.612) | 0.238 | - | - |
| Gender  Male *vs.* Female | 1.071(0.797-1.438) | 0.650 | - | - |
| Tumor depth  T_3_-T_4_ *vs*. T_1_-T_2_ | 2.297(1.559-3.385) | <0.001 | 1.815(1.181-2.789) | 0.007 |
| Lymph node metastasis  N1-N3 *vs*. N0 | 2.657(1.969-3.587) | <0.001 | 2.203(1.543-3.146) | <0.001 |
| Stage  III-IV *vs*. Ⅰ-Ⅱ | 2.628(1.919-3.597) | <0.001 | 1.303(0.864-1.965) | 0.207 |
| *STING* expression  High *vs*. Low | 0.562(0.362-0.873) | 0.010 | 0.636(0.408-0.991) | 0.045 |
