# Supplementary material for: Clinical significance of STING expression and methylation in lung adenocarcinoma based on bioinformatics analysis
Source: Sci Rep. 2022 Aug 17;12:13951. doi: 10.1038/s41598-022-18278-6 (PMC9385651; doi:10.1038/s41598-022-18278-6)
Supplement: Supplementary file 2 — Supplementary Information 2. [file 41598_2022_18278_MOESM2_ESM.zip › Supplementary Information 2/Supplementary Table S9.docx]

**Supplementary Table S9. Univariate and multivariate analysis of the prognostic value of clinical factors and *STING* expression regarding DFS in TCGA LUAD patients.**

| Variables | Univariate analysis | *P*-value | Multivariate analysis | *P*-value |
| --- | --- | --- | --- | --- |
|  | HR (95% CI) |  | HR (95% CI) |  |
| Age  >65 *vs.* ≤65 | 1.270(0.943-1.710) | 0.115 | - | - |
| Gender  Male *vs.* Female | 1.027 (0.766-1.376) | 0.859 | - | - |
| Tumor depth  T_3_-T_4_ *vs*. T_1_-T_2_ | 2.259(1.496-3.410) | <0.001 | 2.286(1.469-3.558) | <0.001 |
| Lymph node metastasis  N1-N3 *vs*. N0 | 1.848(1.372-2.490) | <0.001 | 1.862(1.317-2.631) | <0.001 |
| Stage  III-IV *vs*. Ⅰ-Ⅱ | 1.710(1.198-2.441) | 0.003 | 0.938(0.607-1.451) | 0.774 |
| *STING* expression  High *vs*. Low | 0.665(0.449-0.984) | 0.042 | 0.693(0.467-1.028) | 0.069 |
